# Functional Status of Patients With Schizophrenia Managed in a Community Mental Health Setting in the West Midlands, England

**DOI:** 10.1192/bjo.2026.11734

**Published:** 2026-06-30

**Authors:** Maya De Silva-Morgan, Nilamadhab Kar

**Affiliations:** 1 Black Country Healthcare NHS Foundation Trust, Wolverhampton, United Kingdom; 2 University of Wolverhampton, Wolverhampton, United Kingdom

## Abstract

**Aims:**

Outcomes in schizophrenia vary widely, with a possibility of recovery. Besides symptomatic improvement, functional improvement is a key component of recovery and is a robust outcome indicator in patients with schizophrenia. Improvement in function leads to better self-esteem and quality of life. National Institute for Health and Care Excellence (NICE) guideline CG178 directs providers to document the daytime activities and occupational outcomes for patients with schizophrenia, thus assessing how they are functioning. This work was intended to assess and analyse the documentation of functionality in this patient population during routine clinical appointments.

**Methods:**

This was a clinical audit. Data was collected from digital patient care records including clinic letters. A predesigned questionnaire was utilised for the assessment and documentation of the psychosocial and occupational functioning of 30 consecutive patients with schizophrenia. Each patient had been attending community mental health team clinics for at least one year.

**Results:**

The average age of the sample was 46.0 ± 11.5 years (range 21-67). The mean duration of illness was 15.3 ± 9.8 years (range: 1-42), suggesting that most patients had chronic illness. The cohort included 20 (66.7%) male and 10 (33.3%) female patients, from a range of ethnic backgrounds: 36.7% Caucasians, 26.7% Asians, 20.0% Afro-Caribbean, and others. In routine clinical practice, the assessment and documentation of variables indicating the functional status of patients with schizophrenia were extremely varied and, overall, appeared inadequate. There were no specific scales used to assess patient functioning. Most of the patients had functional impairments in various domains including work, leisure activities, relationships, activities of daily living, and social interaction. However, there were examples of productive engagement with paid and unpaid work in a few patients. Various factors that might contribute to functional impairment were observed, such as physical and mental comorbidities, disabilities, substance misuse, educational underachievement, adverse living conditions and social isolation. In addition, lack of interest in work and refusal to seek employment support were also noted.

**Conclusion:**

Despite available interventions and support, functional impairments in multiple domains continue to impact patients with schizophrenia. There is a need to assess and record the functioning of these patients routinely during clinical interactions. Use of structured and validated metrics may be helpful. In addition to symptomatic improvement, the functionality of the patients is an appropriate indicator of the effectiveness of treatment and degree of therapeutic benefit. Functionality can therefore be used as an outcome measure.

